# Single-pixel three-dimensional imaging with time-based depth resolution

**DOI:** 10.1038/ncomms12010

**Published:** 2016-07-05

**Authors:** Ming-Jie Sun, Matthew P. Edgar, Graham M. Gibson, Baoqing Sun, Neal Radwell, Robert Lamb, Miles J. Padgett

**Affiliations:** 1 Department of Opto-electronic Engineering, Beihang University, Beijing 100191, China; 2 SUPA, School of Physics and Astronomy, University of Glasgow, Glasgow G12 8QQ, UK; 3 Selex ES, Edinburgh EH5 2XS UK

## Abstract

Time-of-flight three-dimensional imaging is an important tool for applications such as object recognition and remote sensing. Conventional time-of-flight three-dimensional imaging systems frequently use a raster scanned laser to measure the range of each pixel in the scene sequentially. Here we show a modified time-of-flight three-dimensional imaging system, which can use compressed sensing techniques to reduce acquisition times, whilst distributing the optical illumination over the full field of view. Our system is based on a single-pixel camera using short-pulsed structured illumination and a high-speed photodiode, and is capable of reconstructing 128 × 128-pixel resolution three-dimensional scenes to an accuracy of ∼3 mm at a range of ∼5 m. Furthermore, by using a compressive sampling strategy, we demonstrate continuous real-time three-dimensional video with a frame-rate up to 12 Hz. The simplicity of the system hardware could enable low-cost three-dimensional imaging devices for precision ranging at wavelengths beyond the visible spectrum.

## Abstract (web summary)

A three-dimensional imaging system which distributes the optical illumination over the full field-of-view is sought after. Here, the authors demonstrate the capability of reconstructing 128 × 128 pixel resolution three-dimensional scenes to an accuracy of 3 mm as well as real-time video with a frame-rate up to 12 Hz.

## Graphical abstract

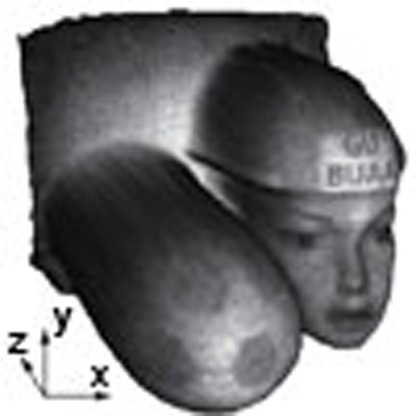

Whilst a variety of three-dimensional (3D) imaging technologies are suited for different applications, time-of-flight (TOF) systems have set the benchmark for performance with regards to a combination of accuracy and operating range. TOF imaging is performed by illuminating a scene with a pulsed light source and observing the back-scattered light. Correlating the detection time of the back-scattered light with the time of the illumination pulse allows the distance *d* to objects within the scene to be estimated by *d*=*tc*/2, where *t* is the TOF and *c* is the propagation speed of light.

The transverse spatial resolution of the image obtained is retrieved either by using a pixelated array or by using a single-pixel detector with a scanning approach^1^,^2^,^3^,^4^,^5^,^6^,^7^,^8^. In both cases, the inherent speed of light demands the use of detectors with a fast response time and high-speed electronic read-out to obtain high precision depth resolution. Advances in sensor development have enabled the first TOF single-photon avalanche detector array cameras to enter the commercial market, having a resolution of 32 × 32 pixels. However, such devices are still in their infancy^9^,^10^,^11^. On the contrary, there are mature single-pixel detectors on the market, which provide stable time-resolved measurements, and by using compressed sensing principles for image reconstruction, which takes advantage of the sparsity in natural scenes, the acquisition times of the scanning approach is largely reduced^12^,^13^,^14^,^15^,^16^.

Recently there have been some interesting developments in 3D imaging utilizing single-pixel detectors. One technique utilizes structured illumination and spatially separated photodiodes to obtain multiple images with different shading properties from which 3D images can be reconstructed via photometric stereo^17^. Another scheme scans a scene, pixel by pixel, using a pulsed illumination source and measures the reflected light using an avalanche photodiode, whereon the first detected photon is used to recover depth and reflectivity via TOF^18^. An alternative method for scanning a scene and recovering depth and reflectivity via TOF has also been demonstrated utilizing structured pulsed illumination^19^,^20^,^21^,^22^.

Among the mentioned demonstrations, many^5^,^18^,^20^,^21^,^22^ used photon-counting detection (that is, Geiger mode), which is well suited for low-light-level imaging. However, one limitation of photon-counting detectors is the inherent electronic dead time between successive measurements, often 10s of nanoseconds, which prohibits the retrieval of short-range timing information from a single illumination pulse. Instead, an accurate temporal response from a 3D scene requires summing the data over many back-scattered photons and hence many illumination pulses (usually several hundreds or thousands^21^,^22^). In contrast, as first demonstrated by Kirmani *et al*.^19^, a high-speed photodiode can retrieve the temporal response from a single illumination pulse, which can be advantageous in certain circumstances, for instance when the reflected light intensity is comparatively large. Incidentally, photon counting cannot operate under such conditions since the detection will always be triggered by back-scattered photons from the nearest, or most reflective, object, rendering more distant objects invisible (Supplementary Note 1).

In this paper, we present a single-pixel 3D imaging system using pulsed structured illumination and a high-speed (short response time) photodiode for sampling the time-varying intensity of the back-scattered light from a scene. We show that by using an analogue photodiode to record the full temporal form of the back-scattered light, along with our original 3D reconstruction algorithm, it is possible to recover surface profiles of objects with an accuracy much better than that implied by the finite temporal bandwidth of the detector and digitization electronics. At distances of ∼5 m we demonstrate a range profile accuracy of ∼3 mm with image resolutions of 128 × 128 pixels, whilst simultaneously recovering reflectivity information of the object. This accuracy is achieved despite a detection bandwidth and a digitization interval corresponding to distances of 150 and 60 mm, respectively. We further demonstrate that by using a compressive sampling scheme, the system is capable of performing continuous real-time 3D video with a frame-rate up to 12 Hz.

## Results

### Experimental set-up

**Figure 1:**
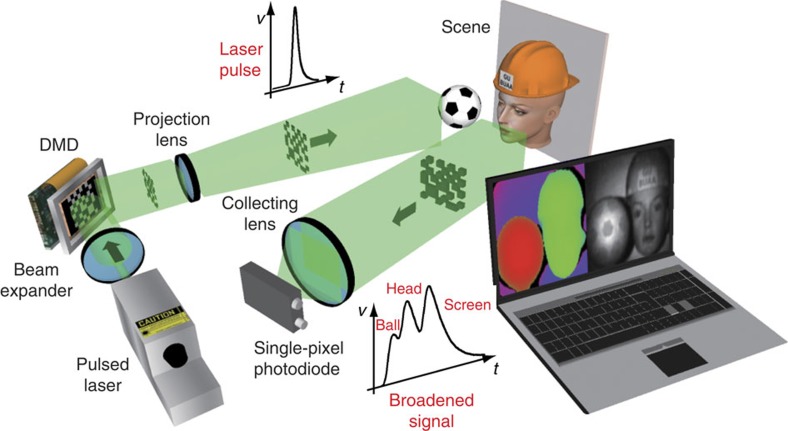
Single-pixel 3D imaging system. A pulsed laser uniformly illuminates a DMD, used to provide structured illumination onto a scene, and the back-scattered light is collected onto a photodiode. The measured light intensities are used in a 3D reconstruction algorithm to reconstruct both depth and reflectivity images.

The single-pixel 3D imaging system, illustrated in Fig. 1, consists of a pulsed laser and a digital micromirror device (DMD) to provide time-varying structured illumination. A high-speed photodiode is used in conjunction with a fresnel lens condenser system to measure the back-scattered intensity resulting from each pattern. The analogue photodiode output is passed through a low-noise amplifier and sampled using a high-speed digitizer. In our work we chose to use the Hadamard matrices^23^ for providing structured illumination. To remove sources of noise, such as fluctuations in ambient light levels, we obtain differential signals by displaying each Hadamard pattern, followed by its inverse pattern, and taking the difference in the measured intensities^24^,^25^. Detailed information about experimental set-up is provided in the Methods section.

**Figure 2:**
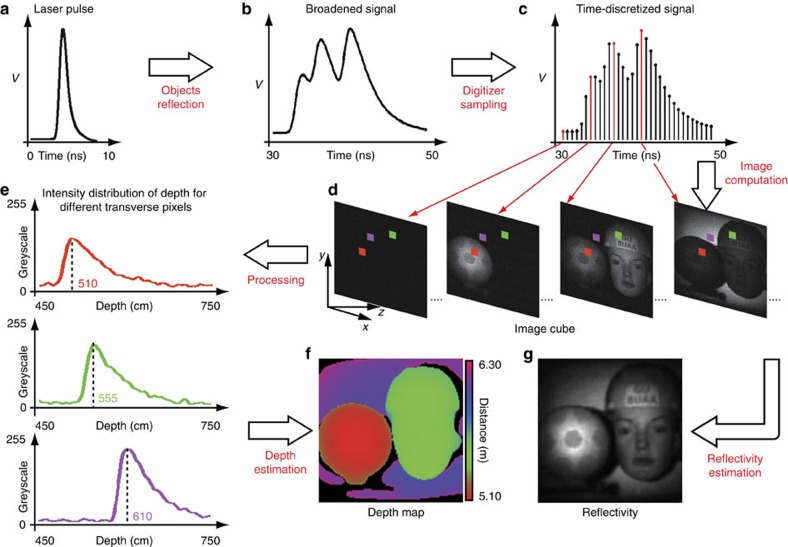
Overview of the reconstruction algorithm. The incident laser pulses (**a**) back-scattered from a 3D scene are temporally broadened (**b**) and discretely sampled using a high-speed digitizer (**c**). An image cube (**d**) is obtained using a reconstruction algorithm, having an intensity distribution along the longitudinal axis (**e**), from which the depth map (**f**) and the reflectivity (**g**) of the scene can be estimated.

A 3D image of the scene is reconstructed utilizing the time-varying back-scattered intensities (measured for each output pulse of the laser) and the associated set of *N* patterns used to provide the structured illumination. An overview of the reconstruction algorithm is shown in Fig. 2 (the result in this diagram represents a scene of three objects ∼0.5 m apart in depth). An incident laser pulse (Fig. 2a) is back-scattered from a scene. The high-speed digitizer converts the amplified analogue signals (Fig. 2b) into discrete data points (Fig. 2c), which are subsequently processed by the computer algorithm. Whereas typical single-pixel imaging schemes use the integrated signal for each illumination pattern to reconstruct a two-dimensional image, our algorithm utilizes *M* discretely sampled intensity points from the time-varying signal to reconstruct *M* two-dimensional images, resulting in an *x*, *y*, *z* image cube (Fig. 2d). In the image cube, each transverse pixel (*x*, *y*) has an intensity distribution (Fig. 2e) along the longitudinal axis (*z*), which is related to the temporal shape of the pulse, the detector response, the read-out digitization and the pixel depth and reflectivity information.

To enhance the range precision beyond the limits imposed by the sampling rate of the system, methods such as parametric deconvolution^19^,^21^ and curve fitting can be used. However, often these methods can be computationally intensive, which makes them unsuitable for real-time applications. Instead we choose to apply cubic spline interpolation to the reconstructed temporal signal at each pixel location, which introduces minimal computational overhead. The depth map of the scene (Fig. 2f) is subsequently determined by finding the maximum in these interpolated signals. In addition, the scene reflectivity (Fig. 2g) can be calculated by averaging the image cube along the longitudinal axis. Utilizing both the depth and reflectivity information, a 3D image of the scene is then reconstructed.

**Figure 3:**
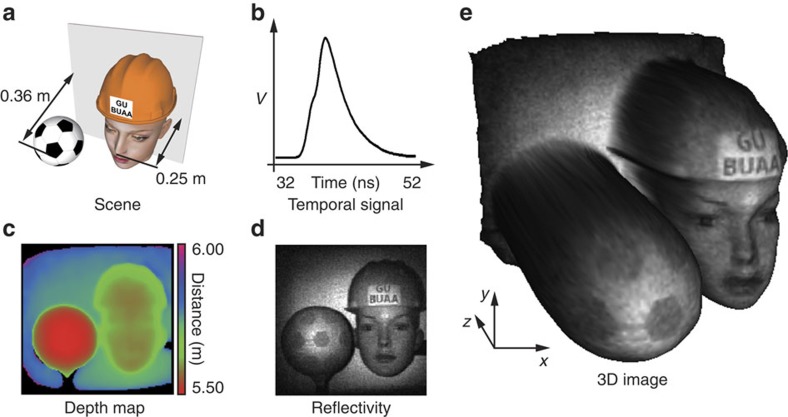
3D image of a scene. (**a**) Illustration of the scene containing multiple objects in close proximity. (**b**) Reflected intensity measured for uniform illumination, indicating temporally indistinguishable objects. (**c**) The estimated depth map of the scene. (**d**) The reconstructed scene reflectivity. (**e**) A textured 3D image of the scene.

**Figure 4:**
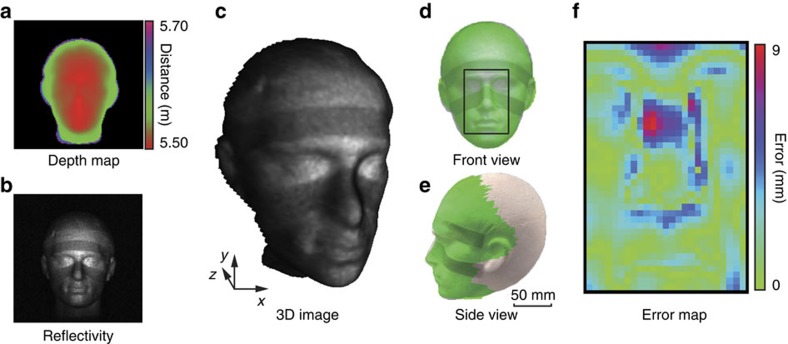
Quantitative analysis of 3D reconstruction. The depth estimation (**a**) reflectivity (**b**) and 3D reconstruction (**c**) of a white polystyrene mannequin head at a range of ∼5.5 m. Superposed depth reconstruction and photograph of the mannequin head, viewed from the front (**d**) and side (**e**). For a chosen region of interest an error map (**f**) showing the absolute differences between our depth result and that obtained using a stereophotogrammetric camera system.

**Figure 5:**
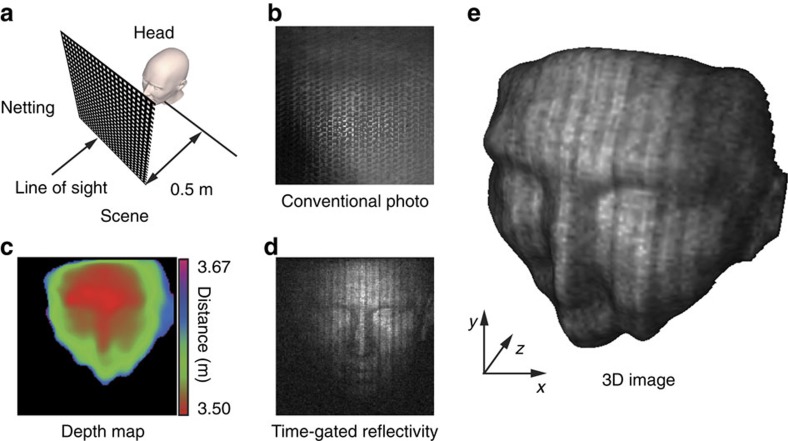
3D imaging through obscuring material. (**a**) Illustration of scene containing a mannequin head with black netting material obscuring the line-of-sight. (**b**) A photograph of the scene taken from the perspective of the 3D imaging system. The scene depth (**c**) and reflectivity (**d**) reconstructed by time-gating the measured intensity data. (**e**) 3D reconstruction of the mannequin head.

**Figure 6:**
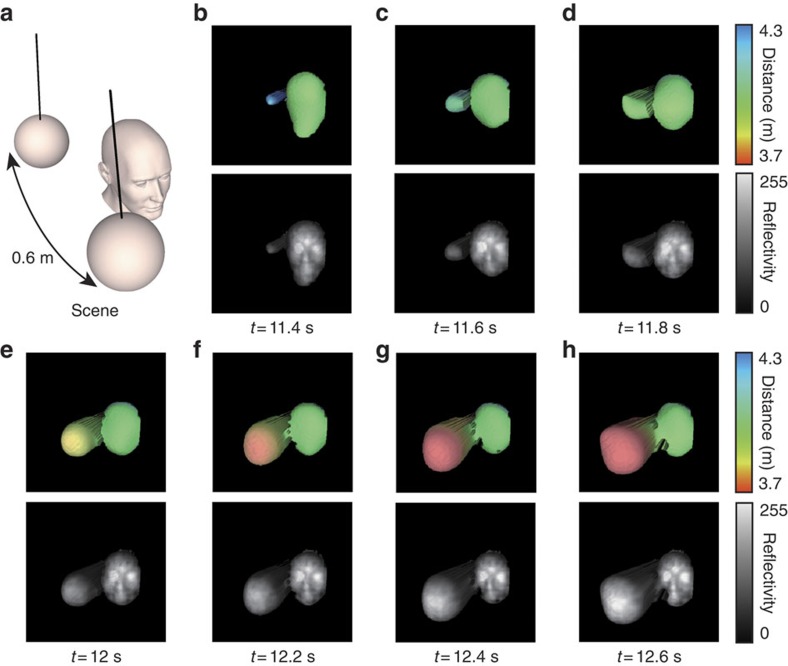
Real-time 3D video. (**a**) Illustration of the scene containing a static mannequin head and a swinging ball. (**b**–**h**) Sample of consecutive depth and reflectivity frames reconstructed at ∼5 Hz frame-rate in real-time for a transverse resolution of 64 × 64 pixels. The experiments of Figs 3, 4, 5, 6 were replicated more than 10 times.

It is worth mentioning that, with the assumption that there is only one surface at each transverse pixel location, our depth estimation (see detail in Methods) works well for scenes that have smooth features, such as the mannequin head and ball, since the reconstructed temporal signals should be slowly varying between sample points. We note that the depth accuracy is limited by the amplitude noise of the data points, over-interpolation only increases the depth precision, but not necessarily the accuracy. In addition, more interpolation adds more processing time, therefore, in our experiments we chose to interpolate by a factor of five times when investigating the static scenes with 20 pulses per pattern (Figs 3, 4, 5), and four times when investigating scenes with motion (Fig. 6), balancing the computational overhead and 3D image quality.

### High-resolution imaging

In one experiment, a scene containing a 140-mm diameter polystyrene soccer ball, a life-size skin-tone mannequin head and a screen was located at a distance of ∼5.5 m from the imaging system (Fig. 3a). The objects were closely separated in distance such that the total depth of the scene was ∼360 mm. A complete Hadamard set of 16,384 patterns, and their inverse, was used as the structured illumination, and the back-scattered intensities (Fig. 3b) measured for reconstructing 128 × 128-pixel resolution depth map, reflectivity and 3D image (Fig. 3c-3e). The illumination time of each pattern was 2.66 ms, corresponding to 20 laser pulses. The total time for acquisition, data transfer from the digitizer buffer to the computer and image processing was ∼130 s. The 3D reconstruction shown in Fig. 3e exhibits distinguishable features, such as the profile of the head and the ball.

To quantitatively determine the accuracy of our 3D imaging system, the scene was modified to contain only a polystyrene mannequin head (180 × 270 × 250 mm), for which we had reference 3D data obtained via a high-accuracy stereophotogrammetric camera system^17^,^26^. The mannequin head was located at a distance of 5.5 m from the imaging system. To further demonstrate the system capability for retrieving reflectivity in addition to the depth, two grey stripes were placed on the head. Performing the same acquisition and imaging processing used in Fig. 3, we obtained the results shown in Fig. 4a–c. Figure 4d,e show the front and side view comparisons between our 3D reconstruction (green) and photographs of the head (white), respectively. After lateral and angular registration and subsequent depth scaling, an error map representing the absolute differences for a chosen region of interest was obtained (shown in Fig. 4f). From this comparison we find our single-pixel 3D imaging system has a root mean squared error of 2.62 mm. More detailed analysis is provided in Supplementary Fig. 1 and Supplementary Note 2.

### Time-gated imaging

One advantage of time-resolved imaging is the ability to distinguish objects at different depths, by artificially time-gating the measured intensity. In certain cases this enables obscuring objects to be isolated from objects of interest. Similar to previous demonstrations^20^, we constructed a 3D scene containing a polystyrene mannequin head (located at a distance of ∼3.5 m) and black-coloured netting used to obstruct the line of sight (located at a distance of ∼3 m), as illustrated in Fig. 5a. An image of the scene taken using a conventional camera is shown in Fig. 5b, where the head is obscured by the black netting. Performing the same acquisition and imaging processing used in Figs 3 and 4, along with an artificial gating on the photodiode data to ensure no reflected signals from the black netting are included in the 3D reconstruction, we obtained the results shown in Fig. 5c–e. As before, we note the characteristic features of the mannequin head can be resolved.

### Real-time compressed video

In addition to obtaining high-quality 3D images of static scenes, many applications demand video frame-rates for motion tracking in dynamic scenes. A key merit of single-pixel imaging is the ability to take advantage of the sparsity of the scene and use compressive sensing to reduce the acquisition time. Most compressive-sensing schemes are performed by minimizing a certain measure of the sparsity, such as *L*_1_-norm, to find the sparsest image as the optimal reconstruction of the scene. However, for resolutions greater than 32 × 32 pixels, the time taken by the construction algorithm often prohibits real-time application^22^,^27^.

In this work we use an alternative scheme, known as evolutionary compressive sensing^6^,^7^, which aims to reconstruct the image with significant less time than conventional compressive sensing by performing a linear iteration (Supplementary Figs 2 and 3 and Supplementary Note 3). In short, the evolutionary compressive sensing scheme chooses a subset of the Hadamard basis to display, by selecting the patterns with the most significant intensities measured in the previous frame, in addition to a fraction of randomly selected patterns that were not displayed. In this experiment, a scene consisting of a static polystyrene mannequin head and a polystyrene white ball (140 mm diameter) swinging along the line of sight with a period of ∼3 s (Fig. 6a). The scene was located at a distance of ∼4 m from the imaging system. Two laser pulses were used per illumination pattern. With the approach described above we obtained continuous real-time 3D video with a frame-rate of 5 Hz using 600 patterns (including their inverse) from the available 64 × 64 Hadamard set, equivalent to a compression ratio of ≃7% (Supplementary Movie 1). The experimental parameters for this result were chosen to balance the inherent trade-off between frame-rate and image quality (Supplementary Note 3). Figure 6b–h show a sample of consecutive frames from the 3D video. The result shows an identifiable 3D image of the mannequin head and ball, in addition to the real-time motion of the ball. Importantly, however, 3D reconstruction can be performed using fewer patterns to achieve higher frame-rates if required, for instance using 256 patterns provides 12 Hz video (Supplementary Movie 2).

## Discussion

We have demonstrated that our single-pixel 3D imaging system is capable of reconstructing a scene with millimetric ranging accuracy using modest hardware. In addition, we obtained real-time video rates by taking advantage of a modified compressive sensing scheme that does not rely on lengthy post-processing.

The performance of the system in this work was mainly limited by the repetition rate of the laser used, 7.4 kHz. Using a laser with a repetition rate greater than or equal to the DMD modulation rate, could enable faster 3D video rates by a factor of three and/or increase reconstruction accuracy by increased averaging.

Furthermore, the broad operational spectrum (400–2,500 nm) of the DMD could enable the system to be extended to the non-visible wavelength, such as the infrared, using modified source and detection optics. The use of DMDs in the infrared has already been demonstrated in microscopy^6^ and real-time video cameras^7^. The potential application of 3D imaging in the infrared could provide enhanced visibility at long-range, due to reduced atmospheric scattering^28^.

## Methods

### Hardware specifications

The following components were used in the experimental set-up (Fig. 1): a pulsed laser (Teem Photonics SNG-03E-100, 532 nm); a DMD (Texas Instruments Discovery 4100 DMD); a projection lens (Nikon ED, *f*=180 mm); a collection lens (customized fresnel condenser lens, *f*=20 mm); a Si biased photodiode (Thorlabs DET10A); and a high-speed USB digitizer (PicoScope 6407, 2.5 GS s^−1^ for two-channel acquisition).

### Operating configurations

There are several important points worth mentioning. (a) The modulation rate of the DMD can reach up to 22.7 kHz, however, in this experiment the DMD is operated in slave-mode, meaning the modulation rate is determined by the repetition rate of the laser at 7.4 kHz. (b) The active area of the photodiode is 0.8 mm^2^, used in conjuction with a 20-mm focal length fresnel lens system, giving a 2.6° field of view, which matches that of the projection system. The depth estimation includes Gaussian smoothing, intensity calibration, cubic spline interpolation and depth determination. More experiment methods and details are provided in Supplementary Methods.

### Data availability

The data used to generate all of the figures in this study can be found at http://dx.doi.org/10.5525/gla.researchdata.317.

## Additional Information

**How to cite this article:** Sun, M.-J. *et al*. Single-pixel three-dimensional imaging with time-based depth resolution. *Nat. Commun.* 7:12010 doi: 10.1038/ncomms12010 (2016).

## Supplementary Material

Supplementary Information Supplementary Figures 1-3, Supplementary Notes 1-3, Supplementary Methods and Supplementary References.

Supplementary Movie 1 Real-time three-dimensional video of a mannequin head and a swinging ball.

Supplementary Movie 2 Real-time three-dimensional video of a swinging ball.
