# Supplementary material for: Single-pixel three-dimensional imaging with time-based depth resolution
Source: Nat Commun. 2016 Jul 5;7:12010. doi: 10.1038/ncomms12010 (PMC5512623; doi:10.1038/ncomms12010)
Supplement: Supplementary Information — Supplementary Figures 1-3, Supplementary Notes 1-3, Supplementary Methods and Supplementary References. [file ncomms12010-s1.pdf]

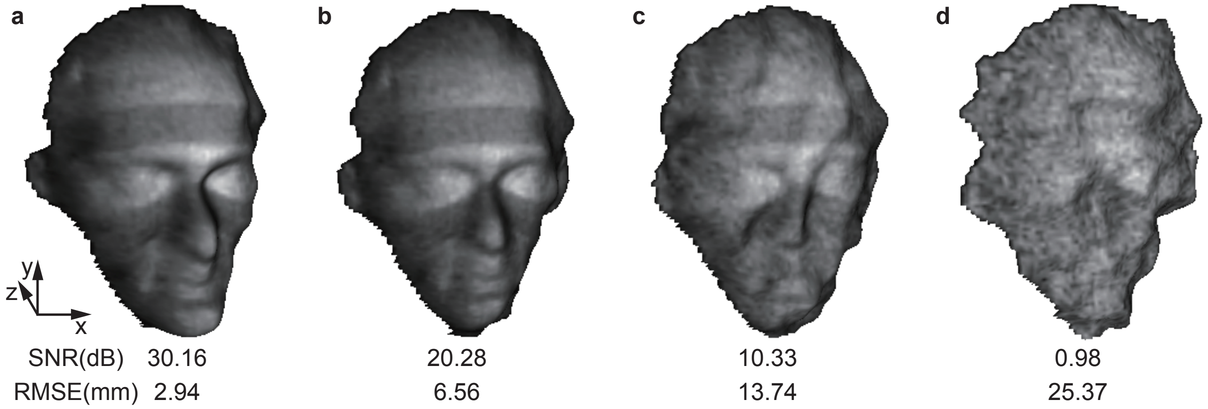

Supplementary Figure 1: System performance with different noise levels. The SNRs are the mean SNR of all measured signals corresponding to the illumination patterns. The root mean square errors (RMSE) are calculated by comparing the experimental results to the data captured by a stereophotogrammetric camera system.

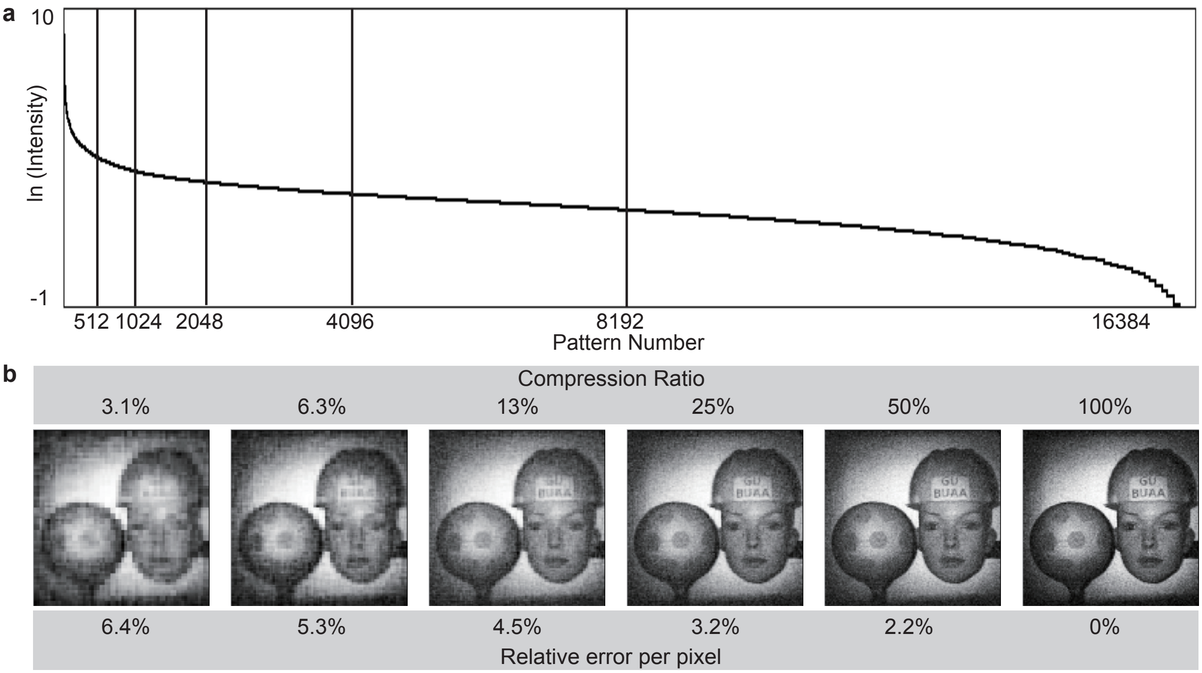

Supplementary Figure 2: Image reconstruction with compression. (a) Ordered Hadamard spectrum, representing the mean temporal intensities measured for a 3D scene. (b) Scene reflectivity reconstructed with different compression ratios, indicating the relative error compared to the reconstruction utilising complete Hadamard basis (100% compression ratio).

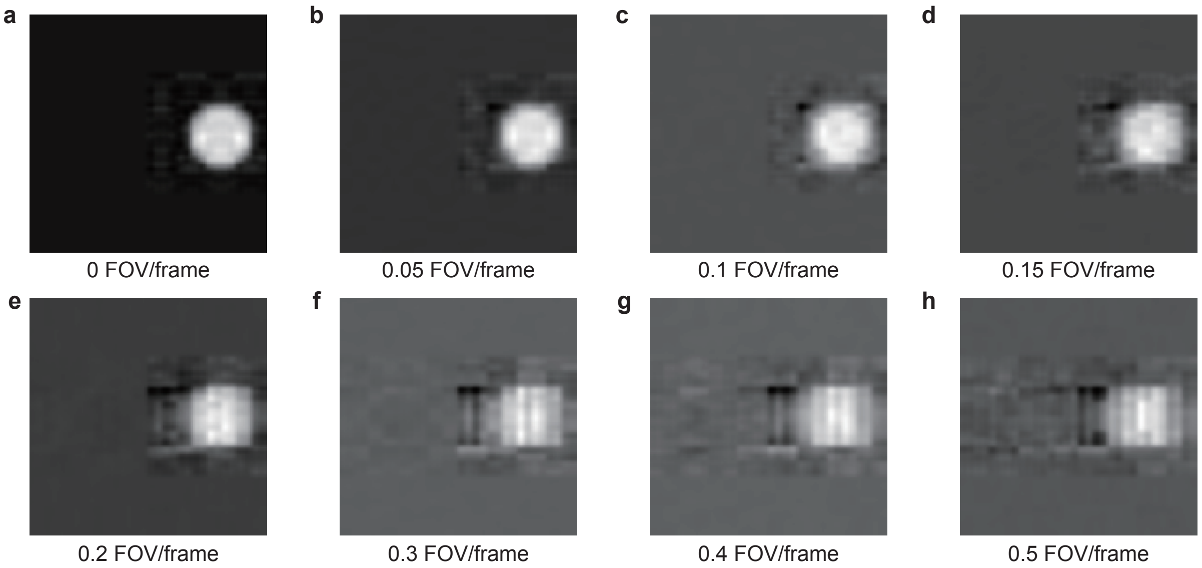

Supplementary Figure 3: One frame reconstruction of different moving velocities. A white ball moves horizontally with different velocities from right to left during the formation of one frame. The compression ratio is $\sim 7\%$. When the ball moves faster than 0.2 FOV/frame, the reconstruction becomes unacceptable.

Supplementary Note 1: TOF photon detection fundamentals

The question of whether a TOF adapted single-pixel imaging system should use a conventional photodiode or one operated with a higher reverse bias, for example an avalanche photon detector (APD), is dependent on the application. A Geiger-mode APD, which can resolve single-photon arrival with a faster response time, is a typical choice for photon counting measurements. Moreover, it is well suited for performing ultra-low light level detection. However, the inherent dead-time of APDs, which is the hold-off time following each counting event, limits the number of counted photons per laser pulse, reflected from objects in close proximity. This requires hundreds of laser pulses per pattern to yield sufficient statistics for recovering accurate depth and reflectivity. In photon counting, the depth resolution is determined by $d_{APD} = D/(2\sqrt{n})$, where D is the pulse width of the laser, n is the number of counted photons in one pattern illumination and the factor of 2 accounts for the outgoing and reflected light. For example in previous work, a 600 mm laser pulse width and collecting 1000 photons per pattern, yielded a depth resolution of ~ 10 mm [1].

In contrast, for a standard photodiode, all photons arriving at the detector contribute to the total time-varying intensity measured, for which the detector response is determined by its bandwidth. In this work we have demonstrated that with just one, or a few, pulse(s) per pattern, accurate 3D images can be reconstructed. However, as the photodiode sensitivity is noise limited, this demands higher reflected light-levels. The depth resolution of this approach is determined by many factors which can be difficult to characterize independently. However, the overall accuracy can be evaluated by using the signal-to-noise ratio (SNR) of the reconstruction. Performing an analysis on the results shown in Fig. 4, we obtain a mean SNR of ~ 38 dB, which predicts a depth resolution of ~ 3 mm, in close agreement with the measured root mean square error (RMSE) of 2.62 mm.

Supplementary Note 2: Quantitative analysis

To quantify the accuracy of our system, we compare the depth map of the polystyrene mannequin head with that captured by a stereophotogrammetric camera system. The latter system uses a matching algorithm on the 2D images from multiple cameras to reconstruct the height map of an object, its accuracy with facial shapes has a RMSE of 1 mm for central facial features and 20 mm at side locations [2]. Therefore we chose to compare a central region of the mannequin head containing the main facial features. After lateral and angular alignment between the two depth maps, the RMSE of our single-pixel 3D imaging system was calculated as 2.62 mm.

We also 3D reconstruct the polystyrene mannequin head to determine how the system performs under the influence of noise. Supplementary Figure 1 shows the 3D reconstruction results with different noise levels. The SNRs listed in Supplementary Figure 1 are the average SNR of all photodiode measured signals for one reconstruction, defined as

$$SNR = 20 \log_{10} \frac{\bar{A}_{signal}}{\bar{\sigma}_{noise}}, \quad (1)$$

where $\bar{A}_{signal}$ is the mean amplitude of the maximum in each measured signal and $\bar{\sigma}_{noise}$ is the mean of the noise standard deviation in each measured signal. The RMSEs listed in Supplementary Figure 1 are obtained using the same method described. Note that the influence of noise on the system performance is not as straight forward as for a pixelated camera, where noise is directly added to the pixel intensities. In single-pixel imaging systems,

different illumination patterns leads to different signal amplitudes while the systematic noise level is more or less the same. Noise has a larger effect on the signals with smaller amplitudes, which usually contribute to image details in the reconstruction. Therefore, as we can see in Supplementary Figure 1, the detailed facial features of the 3D reconstruction degrades significantly as the noise level increases while the outline of the head doesn't change too much.

It is worth mentioning that when the noise level is low (Supplementary Figure 1a), the system performance is quite consistent for consecutive reconstructions. However, when the noise level is high (Supplementary Figure 1c or d), details of the result change dramatically from one reconstruction to another, Supplementary Figure 1c & d each shows only one example.

Supplementary Note 3: Evolutionary compressive sensing

The fundamental idea of evolutionary compressive sensing [3, 4] is to project only a fraction of patterns from the complete set based on the measured intensities of the patterns displayed in the previous frame. In addition to this, an arbitrary fraction of patterns can be chosen at random from the remaining pattern library. This method enables the fraction of patterns with the largest contribution to the image reconstruction to gradually evolve during operation, ideal for dynamically changing scenes. The compression ratio is determined by the total number of patterns chosen to be used, compared to the complete set. Supplementary Figure 2 shows the trade-off between the image quality and the compression ratio.

Two 3D videos (Supplementary Movie 1 & 2) with frame rates of 5 Hz and 12 Hz respectively are obtained using this method. To balance the inherent trade-off between frame-rate and image quality, the compression ratio and the proportion of randomly chosen patterns were tuned depending on the object movement (not the absolute value of the scene velocity but the relative velocity to the system field-of-view (FOV)). For example, in Supplementary Movie 2, the ball swung from side to side with a period of 3 s, and the relative velocity of the ball is ∼0.67 FOV/s. Movie 2 is 12 Hz, thus the ball moved ∼0.05 FOV during the formation of one frame. In this experiment, we manually tuned the compressive ration to ∼7% and the proportion of randomly chosen patterns to 10%, which we think reaches a balance between frame-rate and image quality. It is worth mentioning that, using the same parameters, the system performance varies according to the complexity of the scene and the relative size of the object to the scene. Based on our experience of many experiments with different configurations, generally speaking, the system performance is acceptable when the object moves ∼0.2 FOV/frame. To provide a demonstration of the relationship between the system performance and the scene motion, without other affecting aspects, a simulation with one frame reconstruction of a ball moving at different velocities is added, the simulation result is coincident with our experience.

Supplementary Methods

Illumination patterns

In this work, we take rows/columns from symmetric Hadamard matrices [5] and transform each row/column into a 2D pattern to provide structured illumination. We choose symmetric Hadamard matrices for the following reasons:

(i) All rows/columns of the Hadamard matrices are orthogonal to each other and the orthogonal basis is efficient at sampling signals, which allows us to sample the image with less patterns. (ii) Each row/column is 50% 'on' and 50% 'off' and therefore provides illuminations with a constant light-level and self-normalized signals, which improves the resulting SNR [6, 7]. (iii) The inverse of a symmetric Hadamard matrix is its own (multiplying a constant), it enables us to reconstruct the image with a linearly iterative algorithm, which requires very low computational complexity.

Differential signals

During the development of single-pixel imaging, a number of algorithms, known as differential ghost imaging [7–10], have been developed to improve the SNR of the results. In this work, one differential signal is obtained by displaying each Hadamard pattern, followed by its inverse pattern, and taking the difference between the two measured intensities. This procedure can be viewed as a binary version of differential ghost imaging, which follows the same physical principle and achieves similar improvement on SNR.

Depth calibration

The acquisition of the PD signals is triggered when each laser pulse is emitted. The TOF is the time delay between triggering and the arrival of the back scattered intensity, which determines the corresponding depth. Note that this depth is only accurate once electronic delay has been calibrated. To this end a white screen was placed at different known distances and a fixed temporal/depth offset was required to perform calibration.

Image cube reconstruction

In single-pixel imaging based on projected patterns, the intensity signal S_i associated with each projected pattern P_i, is directly proportional to the overlap between the scenes reflectivity distribution and the pattern. The patterns are generated from an orthogonal basis, such that an M pixel image can be fully sampled after performing M measurements and linear projections, and an image I_{2D} can be reconstructed as $I_{2D} = \sum_{i=1}^{M} S_i \cdot P_i$.

In our single-pixel 3D imaging system, the intensity signal $S_i(d)$ for any pattern is determined by the scene reflectivity at different depths $d = ct/2$, where c is the speed of light and t is the time-of-flight. The role of the digitizer is to perform discrete samples of the intensity signal, which enables an image cube, I_{3D}, of the scene to be reconstructed by $I_{3D}(d) = \sum_{i=1}^{M} S_i(d) \cdot P_i$. Hence, the image cube is a discretized array of 2D images.

Depth estimation

For our system the digitizer sampling rate is faster than the photodiode response, which results in objects within the image cube appearing at multiple depths, thus in order to recover accurate depth, the following protocol was developed:

- **Gaussian smoothing**. In order to denoise the reconstructed images at different depths and avoid false depth detection, a Gaussian kernel is applied in a local linear regression algorithm to smooth these images one by one

[11].

- **Intensity calibration**. According to energy conservation, the decay of back scattered intensity is proportional to the square of the distance between the target and detector. Therefore, the intensities of the ith image in the cube is multiplied a factor of $(d_i/d_1)^2$, where d_i is the depth of the ith image, d_1 is the depth of the first image in the cube.

- **Cubic spline interpolation**. The depth interval between each image in the cube is determined by the sampling rate of the analogue-to-digital conversion. For our system, the time interval between each sample point is 0.4 ns, which means the depth interval of the image cube is 60 mm. However, using spline interpolation, we can transform the image cube into a denser one. In Figs. 3-5, we apply the interpolation by a factor of 5 times, which generates 31 points between two adjacent sampled data points. The depth interval between two points is $\sim$ 2 mm, matching the measured RMSE of 2.62 mm in Fig. 4. In Fig. 6, the interpolation factor is 4 times, thus the depth interval is $\sim$ 4 mm. The cubic spline interpolation guarantees that the first and second derivatives of the cubic interpolating polynomials are continuous, even at the data points. By applying cubic spline interpolation to the image cube data, the values of generated points can be larger than that of the data points themselves, providing the possibility of interpolating point that are closer to the actual maximum intensity, therefore estimating the depth more accurately.

- **Depth determination**. Assuming an image cube of $m \times m \times l$, where $m \times m$ is the pixel resolution of an image and l is the number of images the cube contains, there is a longitudinal intensity array of length l. With the assumption that there is only one surface at each transverse pixel location, we chose to use the depth whereupon the reflected intensity was maximum as the depth of the scene for that transverse pixel location.

Supplementary References

---

[1] Howland, G. A., Lum, D. J., Ware, M. R., and Howell, J. C. *Opt. Express* **21**(20), 23822–23837 (2013).

[2] Khambay, B., Nairn, N., Bell, A., Miller, J., Bowman, A., and Ayoub, A. *Br. J. Oral Maxillofac. Surg.* **46**, 27 (2008).

[3] Radwell, N., Mitchell, K. J., Gibson, G. M., Edgar, M. P., Bowman, R., and Padgett, M. J. *Optica* **1**, 285–289 (2014).

[4] Edgar, M. P., Gibson, G. M., Bowman, R. W., Sun, B., Radwell, N., Mitchell, K. J., Welsh, S. S., and Padgett, M. J. *Sci. Rep.* **5**, 10669 (2015).

[5] Pratt, W. K., Kane, J., and Andrews, H. C. *Proceedings of the IEEE* **57**(1), 58–68 (1969).

[6] Sun, B., Welsh, S. S., Edgar, M. P., Shapiro, J. H., and Padgett, M. J. *Opt. Express* **20**, 16892–16901 (2012).

[7] Sun, B., Edgar, M., Bowman, R., Vittert, L., Welsh, S., Bowman, A., and Padgett, M. In *Computational Optical Sensing and Imaging*, OSA, CTu1C–4. (2013).

[8] Ferri, F., Magatii, D., Lugiato, L. A., and Gatti, A. *Phys. Rev. A* **104**, 253603 (2010).

[9] Kai-Hong, L., Bo-Qiang, H., Wei-Mou, Z., and Ling-An, W. *Chin. Phys. Lett.* **29**(7), 074216 (2012).

[10] Sun, M.-J., Li, M.-F., and Wu, L.-A. *Appl. Opt.* **54**(25), 7494–7499 (2015).

[11] Bowman, A. W. and Azzalini, A. *Comput. Stat. Data Anal.* **42**(4), 545–560 (2003).
